# Clinical Pharmacology of Antibody-Drug Conjugates

**DOI:** 10.3390/antib10020020

**Published:** 2021-05-21

**Authors:** Iftekhar Mahmood

**Affiliations:** Mahmood Clinical Pharmacology Consultancy, LLC, Rockville, MD 20850, USA; Iftekharmahmood@aol.com; Tel.: +1-301-838-4555

**Keywords:** ADCs, clinical pharmacology, pharmacokinetics, conjugation, oncology

## Abstract

Antibody-drug conjugates (ADCs) are biopharmaceutical products where a monoclonal antibody is linked to a biologically active drug (a small molecule) forming a conjugate. Since the approval of first ADC (Gemtuzumab ozogamicin (trade name: Mylotarg)) for the treatment of CD33-positive acute myelogenous leukemia, several ADCs have been developed for the treatment of cancer. The goal of an ADC as a cancer agent is to release the cytotoxic drug to kill the tumor cells without harming the normal or healthy cells. With time, it is being realized that ADCS can also be used to manage or cure other diseases such as inflammatory diseases, atherosclerosis, and bacteremia and some research in this direction is ongoing. The focus of this review is on the clinical pharmacology aspects of ADC development. From the selection of an appropriate antibody to the finished product, the entire process of the development of an ADC is a difficult and challenging task. Clinical pharmacology is one of the most important tools of drug development since this tool helps in finding the optimum dose of a product, thus preserving the safety and efficacy of the product in a patient population. Unlike other small or large molecules where only one moiety and/or metabolite(s) is generally measured for the pharmacokinetic profiling, there are several moieties that need to be measured for characterizing the PK profiles of an ADC. Therefore, knowledge and understanding of clinical pharmacology of ADCs is vital for the selection of a safe and efficacious dose in a patient population.

## 1. Introduction

Monoclonal antibodies (mAbs) are widely used therapeutic agents in hematology and oncology. Despite the therapeutic benefit, these antibodies either do not have the desired clinical efficacy or have to be co-administered with traditional chemotherapy. These shortcomings of mAbs led to efforts to enhance the therapeutic benefit of mAbs by modifications, mainly by forming a conjugate [1].

Antibody-drug conjugates (ADCs) are biopharmaceutical products where a monoclonal antibody is linked to a biologically active drug (a small molecule) forming a conjugate [1]. The first ADC Gemtuzumab ozogamicin (trade name: Mylotarg) for the treatment of CD33-positive acute myelogenous leukemia was approved by the USA Food and Drug Administration (FDA) in 2000 [1]. It was however, withdrawn from the market in 2010 due to adverse events particularly hepatic side effects. Mylotarg was reintroduced in the market in 2017 with a lower dose than the previous one and with a new indication acute myeloid leukemia. These days, the pharmaceutical companies are heavily focused on the development of ADCs to treat a wide variety of diseases. Initially, most of the ADCs were developed for the treatment of oncology and hematology, but with time it has been realized that ADCs can also be developed to manage or cure other diseases such as inflammatory diseases, atherosclerosis, and bacteremia [2]. The goal of an ADC as a cancer agent is to release the cytotoxic drug to kill the tumor cells without harming the normal or healthy cells.

There are three components of ADCs: an antibody, a cytotoxic drug (small molecule drug also known as payload), and a specialized chemical linker which connects the mAB with the small molecule [3]. All these three components are critical in designing an ADC. The antibody should be specific for a cell surface target molecule that is selectively or over-expressed on cancer cells compared to normal cells. The payload or the drug should be potent enough to kill tumor cells at intracellular concentrations. Generally there are three to four payloads on a mAb. The tumor cell death occurs either by causing irreversible DNA damage or interfering with the mechanism of cell division. ADCs are designed with linkers that release biologically active drug. The linkers are also very critical components of ADCs. The linkers should be stable enough in the circulation to keep the antibody-drug conjugate intact in the systemic circulation and when distributed in the tissues including tumor it allows to release the active drug in the tumor once the ADC is taken up in the tumor [3]. The efficacy of an ADC depends on its intracellular activities (endocytosis) and processing of its components to cause tumor cell death. The internalized ADCs are degraded to release cytotoxic payloads inside the tumor cell by the cleavage of the linker by specific proteases or by lysosomal process, leading to cell death.

Clinical pharmacology is one of the most important tools of drug development since it helps in finding an optimum dose of a product, thus preserving the safety and efficacy of the product in a patient population. The clinical pharmacology aspects of ADCs are described below.

## 2. Components of ADCs

ADCs are heterogeneous compounds, and several analytes are found in the systemic circulation that can be detected by the available analytical methods. The analytes that are generally measured are: the conjugated antibody or ADC (antibody with drug), the total antibody (conjugated, partially deconjugated and fully deconjugated), the antibody-conjugated drug (the total small molecule drug conjugated to antibody), the unconjugated drug (small molecule drug not conjugated to antibody), and possibly metabolites of the small molecule drug including or not part of the linker [3,4]. Total antibody and total ADC concentrations are measured in relation to efficacy and on-target toxicity, while unconjugated drug concentrations are monitored for potential off-target toxicity.

Due to the limitations of the bioanalytical methods, only certain components of ADCs can be measured and PK analysis on these moieties or analytes can be performed. Generally, PK parameters are determined for total antibody, ADC or conjugated mAb, and unconjugated drug in blood or plasma [3,5]. ADCs are given by intravenous infusion and the typical PK characteristics of ADCs are that ADC and total antibody maximum plasma concentration (C_max_) reaches at the end of infusion and then decline mono- or multi-exponentially, with terminal half-lives ranging from 3 to 20 days. The total antibody concentrations in serum or plasma are higher than those of the ADC. The volume of distribution is either close to or slightly greater than blood or plasma volume. The unconjugated analyte follows a formation limited kinetics and is at much lower concentrations than the total antibody or ADC [3,5].

## 3. Clinical Pharmacology of ADCs

### 3.1. Pharmacokinetics

Pharmacokinetics (PK) is an integral part of clinical pharmacology and the modern-day drug development process. PK is a quantitative analysis of how living systems handle xenobiotics. Pharmacokinetics is the study and characterization of the time course of drug absorption, distribution, metabolism, and excretion (ADME) [6]. In pharmacokinetics, mathematical models are used to describe the rate processes of drug absorption, distribution, and elimination. Through these mathematical models, equations can be developed, which can then be used to describe drug concentrations in the body as a function of time [6].

The main objective of a pharmacokinetic study is to obtain information regarding absorption, volume of distribution, clearance (renal and metabolic), half-life, accumulation of a drug after multiple dosing, and the effects of various disease states (renal and/or hepatic) as well as age, weight, and gender on the pharmacokinetics of a drug. These pharmacokinetic parameters can then be used to design an optimal dosing regimen in a patient or initiate phase II and phase III clinical trials [6]. The PK parameters can also be linked to the time course of pharmacological response (therapeutic and/or toxicologic) known as ‘pharmacodynamics’ [7].

Several computer programs are available for the analysis of plasma concentration versus time data to estimate pharmacokinetic parameters. Computer programs are helpful in characterizing the pharmacokinetics of a drug in terms of a model-dependent (compartmental PK) approach. With the availability of model-independent (non-compartmental analysis) methods, there is not much interest in a model-dependent approach. However, modeling of pharmacokinetic data remains an important feature of pharmacokinetic analysis. Pharmacokinetic models can be used to [6]:

- predict plasma, blood, and tissue concentrations of a drug
- calculate the optimum dose in a patient
- describe how changes in physiology or disease state can affect the pharmacokinetics of a drug
- correlate drug concentrations with pharmacologic or toxicologic response
- calculate the accumulation of a drug following multiple dosing

It should be recognized that unlike small molecules and therapeutic proteins (antibodies and non-antibodies), the PK of an ADC is very complex because an ADC consists of several components. One not only has to consider the PK of the mAb but also the PK of the cytotoxic molecule as well as the physicochemical properties of the binding. The PK of different components of an ADC is greatly affected by the PK of the mAb because it represents more than 90% of the molecular weight. The PK profile of the total mAb (ADC + mAb alone) provides the best estimate of the stability and integrity of the ADCs. The conjugation and the site of conjugation also plays an important role in maintaining the stability and the PK of ADCs [8,9,10]. The characteristics of the FDA-approved ADCs and their PK are described in Table 1 and Table 2.

### 3.2. Estimation of PK Parameters

From blood or plasma concentrations versus time data, PK parameters of a drug (small and large molecules) can be estimated. The principles of PK are applicable and are similar across the class of drugs (small and large molecules). The PK parameters can be obtained either by compartmental (model-dependent) or non-compartmental (model-independent) analysis [6,14].

### 3.3. Compartmental Analysis

A compartment is not a real physiologic or anatomic entity; rather, it is considered as a tissue or group of tissues that have similar blood flow and drug affinity. For example, the heart, lung, kidneys, brain, and liver are well-perfused with blood. As a result, drugs distribute quickly and uniformly into these organs; hence, the aforementioned tissues can be lumped as a single compartment. Drug molecules freely move in and out of compartments and rate constants can be used to describe the overall rate processes of drugs in the body. Thus, in a compartment model, one can group tissues into several compartments based on the blood flow and drug affinity. The compartments are generally referred to as the central and peripheral compartment. Blood as well as rapidly perfused organs (heart, lung, kidneys, brain, and liver) constitute the central compartment. Muscles and fat are examples of peripheral compartments. The central compartment is the only one that is directly accessible for sampling. Plasma concentrations versus time data can be described either by a single- or multi-compartment model [6,14]. In fact, the compartmental model is similar to a minimal physiologically based pharmacokinetic (PBPK) model.

Although non-compartmental analysis is preferred over compartmental analysis, it is important to model the extensive sampling data in phase I studies. The modeling approach will yield useful quantitative information about the drug and can be used for simulation, prediction of PK parameters at steady state and later for population PK studies when only a limited number of blood samples is available from the patient population [6,14].

### 3.4. Non-Compartmental Analysis

All models are empirical and modeling can be complex, time consuming, and erratic. Therefore, these days, a simpler and more convenient approach is taken to calculate the pharmacokinetic parameters. This approach, known as ‘non-compartmental analysis’, is based on the statistical moment theory and is independent of the route of administration [14]. Non-compartmental methods do not require any assumption of a specific compartmental model and can in fact be applied to any compartmental model, provided linear pharmacokinetics is assumed [6,14]. From blood or plasma concentrations versus time data, the following PK parameters of a compound (both small and large molecules) can be estimated [6,14].

#### 3.4.1. Maximum Plasma Concentration (C_max_)

C_max_ is the maximum plasma concentration on a concentration–time curve and is dependent on the sampling time. Therefore, a true C_max_ can be different than observed on the concentration–time curve.

#### 3.4.2. Time to Reach Maximum Plasma Concentration (T_max_)

T_max_ is the time to reach C_max_. As with C_max_, T_max_ is also dependent on the sampling time.

#### 3.4.3. Area under the Curve (AUC)

AUC represents the exposure of a drug or a compound in a living system. AUC of a drug concentration versus time plot is calculated from time zero to infinity [6]. The AUC from time zero to infinity (∞) is calculated in two steps. The AUC from zero to time t* (the time of the last measurable concentration) is calculated by the trapezoidal rule and then extrapolated to infinity by adding the area from time t* to infinity. This extrapolated area can be calculated from the following equation:

(1) $$\mathrm{Area}\;\mathrm{from}\;t^{*}\;\mathrm{to}\;\infty =\frac{c^{*}}{k}$$

where c* is the last concentration and k is the elimination rate constant.

The extrapolation of area from the time of the last measurable concentration to infinity should only be carried out when the blood samples have been collected for a sufficiently long period of time. Only 20% of the extrapolated area to the total area is acceptable in PK analysis.

#### 3.4.4. Area under the Moment Curve (AUMC)

AUMC is the total area of a drug concentration versus time plot obtained from the product of drug concentration and time (c × t) versus time from time zero to infinity [6]. As with the AUC, AUMC from time zero to infinity (∞) is also calculated in two steps. The AUMC from zero to time t* (the time of the last measurable concentration) is calculated by the trapezoidal rule and then extrapolated to infinity by adding the area from time c* × t* to infinity. This extrapolated area is calculated from the following equation:

Area from t* to ∞ = (c* × t*)/k + c*/k^2^ (2)

where c* × t* is the product of the last measured concentration and time, and k is the elimination rate constant.

#### 3.4.5. Clearance (CL)

Clearance is the most important pharmacokinetic parameter. The total clearance of a drug from the body involves more than one organ (liver, kidneys, lung, gut, etc.). Total or systemic clearance is the sum of all individual organ clearances responsible for the overall elimination of a drug. Blood or plasma clearance can be defined as the volume of drug cleared from blood or plasma in unit time. Following a single intravenous dose, the clearance (termed as systemic clearance) can be calculated from the following equation:

(3) $$\mathrm{CL}=\frac{\mathrm{Dose}}{{\mathrm{AUC}}_{\left(0-\infty \right)}}$$

#### 3.4.6. Terminal Half-Life

The half-life of a drug is the time required for a 50% decrease in drug concentration and is calculated according to the following equation:

Half-life = 0.693/k(4)

where k is the elimination rate constant.

For the estimation of half-life, it is important to collect long enough blood samples (at least four half-lives). For example, if a drug has a half-life of 12 h, then the blood sample should be collected at least within 48 h.

#### 3.4.7. Mean Residence Time (MRT)

MRT is analogous to half-life but it represents the time required to eliminate 63.2% of the administered dose. The MRT after a single dose can be calculated by the equation:

(5) $$\mathrm{MRT}=\frac{{\mathrm{AUMC}}_{\left(0-\infty \right)}}{{\mathrm{AUC}}_{\left(0-\infty \right)}}$$

#### 3.4.8. Volume of Distribution XE “Volume of Distribution” of Central Compartment (V_c_)

The volume of distribution of the central compartment can be calculated as follows:

(6) $$\mathrm{Vc}=\frac{\mathrm{Dose}}{\mathrm{intercept}}$$

where intercept is the value extrapolated to the *y*-axis.

#### 3.4.9. Volume of Distribution at Steady State (V_ss_)

V_ss_ is the product of clearance and mean residence time, following a single intravenous bolus dose of a drug.

V_ss_ = CL × MRT (7)

## 4. Pharmacokinetic Characteristics of ADCs

Generally, after the administration of a drug in the body, four processes are involved. These processes are absorption, distribution, metabolism, and elimination (ADME), irrespective of the nature of the molecules (small and large molecules); a brief description of these processes is provided below for ADCs.

### 4.1. Absorption

Following the administration of a drug, the drug reaches into the systemic circulation [6]. Generally, the drugs are administered by oral, intravenous, intramuscular, and subcutaneous routes. Each of these methods has its own ADME characteristics, and advantages and disadvantages.

Most of the antibodies are generally given by intravenous (IV) bolus or infusion routes. Antibodies can also be given by subcutaneous (SC) route. However, for ADCs, at the moment, the route of administration is IV or IV infusion. SC administration may not be feasible for ADCs, due to the reactions to cytotoxic payloads and local deposits of cytotoxic material [3,4].

### 4.2. Distribution

Following the administration of a drug, and after absorption, a drug is distributed throughout the body. The distribution of a drug is based on the biochemical properties of the drug. Distribution is a process of convection and diffusion, which is regulated by polarity, size, protein concentrations and the binding abilities of the drug to the tissues [6].

The distribution of a drug in the body can be described by the volume of distribution. Due to their size and polarity, the distribution of antibodies and ADCs is generally confined to the vascular and interstitial space [5].

The initial distribution of ADCs is generally confined to the blood compartment, and the volume of distribution is generally equal to blood volume. Later, ADCs can distribute to the interstitial space.

ADC distribution can also be affected by target antigen expression and internalization [15,16].

The distribution of unconjugated antibodies to non-target tissues, by antigen-specific or antigen non-specific, may have little or no pharmacologic impact. On the other hand, the distribution and accumulation of an ADC to the same tissues can produce undesirable (toxic) pharmacological impact due to the uptake of ADC and subsequent release of cytotoxic drug or the metabolites of the cytotoxic drug [9,10].

### 4.3. Metabolism and Elimination

Metabolism is a chemical process through which a drug is excreted from the body. Metabolism leads to the inactivation of the drug by converting it into a more hydrophilic molecule, which can then be excreted in the urine. The major site of drug metabolism is the liver, but the gastrointestinal tract, kidneys, and lungs can also be the site of drug metabolism [6].

Most of the small molecules either undergo hepatic drug metabolism through phase I and/or phase II reactions or are excreted entirely or partly renally [6]. Phase I reactions are mediated by CYP enzymes, whereas phase II reactions are involved with conjugation pathways (glucuronidation, sulfation, and glutathione) [6].

The metabolism and elimination of macromolecules differ significantly from small molecules. Macromolecules are not eliminated from the body as with small molecules. A complex combination of specific and non-specific mechanisms is involved in the elimination of antibodies and ADCs [17,18,19]. The clearance of ADCs is very much similar to mAbs. The clearance of an ADC is a cumulative process of deconjugation (the loss of drug from the ADC) and the catabolism of the ADC. ADCs are broken down nonspecifically via proteolysis in many tissues such as the liver, kidneys, skin, and muscle. Furthermore, pinocytosis degrades the antibodies via lysosomal enzymes. ADCs can also be cleared by a target-mediated mechanism and this may lead to saturable clearance pathways, resulting in nonlinear PK of antibodies and ADCs [20].

## 5. Bioanalysis

There are several analytes of ADCs, and in order to characterize the PK profiling of these analytes, several analytical methods are required and are described below [17,21,22]:

- ELISA immunoassays measuring the conjugate and total antibody kinetic profiles;
- TFC-MS/MS, which quantifies free drugs/metabolites; and
- High-resolution mass spectroscopy for drug-antibody ratio (DAR) analysis in vivo.

Furthermore, two types of ELISA immunoassays are used for quantitative measurement of analytes of an ADC: the first type of assay measures the total antibody, which is the ADC with a drug-antibody ratio (DAR) higher than or equal to zero. The second type of assay measures the drug-conjugated antibody, defined as the ADC with a DAR greater than or equal to one [17,21,22].

Other analytical methods are size-exclusion chromatography (SEC) and hydrophobic interaction chromatography (HIC). SEC is the most commonly used liquid chromatography (LC) technique to determine the number of aggregates for mAbs, and this technique can also be applied for ADCs [23]. Although, HIC is a traditional technique used for the separation, purification, and characterization of proteins, this technique is now being used for ADC characterization and analysis. Fleming [24] has described a generic HIC protocol for the screening, analysis, and characterization of ADCs using an ammonium sulfate buffer and a high-pressure liquid chromatography system.

## 6. Cytotoxic Payload

Monoclonal antibodies (mAbs) are widely used as therapeutic agents to manage a wide variety of diseases, especially cancer. Due to the limitations of the mAbs antitumor efficacy, many different approaches are being used to modify mAbs to increase the antitumor efficacy. These methods include the conjugation of mAbs to radionuclides, fusion with protein toxins (immunotoxins), or coupling to small molecule drugs (antibody–drug conjugates, ADCs) [25,26]. The coupling of a mAb with a cytotoxic agent or a small molecule is called ‘payload’ [25,26]. The following can be described as the desirable characteristics of a payload.

1. The cytotoxic capability for a payload should be high with proper lipophilicity.
2. The target of the payload should be located inside the cells.
3. The molecular structure of a payload should be small in size, lack immunogenicity, soluble in aqueous buffers so that it can be easily conjugated
4. The payload should be stable in plasma.

The payloads used in ADCs can be divided into two main classes: microtubule inhibitors and DNA-damaging agents [27,28]. Two currently widely used microtubule inhibitors are maytansinoids and auristatins. There are two maytansinoids derivatives: DM1 and DM4. DM1 includes emtansine and mertansine. DM4 includes soravtansine and ravtansine [27]. Other payloads include topoisomerase I inhibitors, which enhance the antitumor immune response. The payload for The payload for sacituzumab govitecan [29] and trastuzumab deruxtecan [30] are topoisomerase I inhibitors [29]. DNA-damaging agents include calicheamicin, pyrrolobenzodiazepines (PBD) dimer, indolinobenzodiazepines, duocarmycins, and doxorubicin [28].

## 7. Drug-Antibody Ratio (DAR) and Conjugation of ADCs

The drug–antibody ratio (DAR) is defined as the average number of payload molecules attached to a single mAb, generally between two and four [31]. In very few cases, a DAR as high as 8 has safely been achieved through the use of hydrophilic linker-payloads [31,32]. The examples are approved ADCs such as Enhertus and Trodelvys. DAR is very important for the determination of efficacy of ADCs. Besides, DAR may affect the drug stability in the circulation, PK, and toxicity of an ADC [31,32].

DAR widely varies and depends on other ADC variables. If there are too few molecules attached to the antibody, then one may not achieve the desired therapeutic benefit or too many molecules attached to the antibody may lead to the instability of the molecule and alter the overall PK and PD characteristics of an ADC [31]. Studies have shown that ADCs with high DAR (7 to 14) values had faster clearance and decreased in vivo efficacy compared with ADCs with a DAR value < 6 [32]. The DAR values and their impact on stability and PK are also dependent on the site of conjugation and the use of lighter or heavier conjugated chains [33].

Lysine or cysteine are generally modified for the creation of protein conjugates. Lysine is one of the most commonly used amino acid residues for linking substrates to antibodies. Lysine is generally found on the surface of the antibody; hence, it is easily accessible. Lysines are generally modified with N-hydroxysuccinimide (NHS) esters, sulfonyl chlorides, isocyanates and isothiocyanates. Mylotargs, Kadcylas and Besponsas all use the lysine bio-conjugation technique and are administered as a heterogeneous mixture. Fifty percent of the mAbs Mylotargs are unconjugated (DAR = 0), with the remaining species averaging DAR = 6, with an overall average DAR of 3 [34,35]

Other amino acids such as cysteine and tyrosine can also be modified, but such modifications lead to the formation of less stable linkages. These less stable linkages can release the linker payload prematurely, causing toxicity and lowering efficacy. Cysteine modification is carried out by 1,4-conjugate addition to N-substituted maleimides [35]. ADCs such Adcetriss, Polivys, Padcevs, Enhertus, Trodelvys and Blenreps are synthesized by maleimide modification of cysteines. All nine of the currently approved ADCs are synthesized via modification of either lysine or cysteine.

N-Terminal transamination is another strategy that can be used to synthesize ADCs in a site-selective manner by converting the amino N-terminus to a ketone or aldehyde group [35].

## 8. Structural Modification

In order to improve the PK characteristics of therapeutic proteins, sometimes structural modifications are carried out. The most common method(s) to modify these agents is by glycosylation or PEGylation. These modifications can be made to the antibody and are intended to reduce the clearance and increase the half-life of therapeutic proteins [36].

### 8.1. Glycosylation

Glycosylation is the modification of therapeutic proteins since glycosylation is important for many ligand-receptor interactions. Glycosylation is basically the addition of certain sugars (glycans) to the protein moiety. Glycans enhance protein stability, regulate the ligand-receptor interaction and protein secretion as well as improve the PK characteristics of the proteins [37].

In a study by Zhou et al. [38], using galactosyl and sialyltransferases altered the native glycans on Asn-297 of antibodies. They applied their glycosylation method to three antibodies including trastuzumab (Ado-trastuzumab emtansine) and two cytotoxic agents. Their approach (glyco-conjugated ADCs) provided greater antitumor activity than antibody without glycosylation in a Her2-positive tumor xenograft model. The impact of glycosylation on the PK, efficacy, and safety on ADCs have not been fully evaluated and further investigation in this direction is needed.

### 8.2. Pegylation

Attachment of polyethylene glycol (PEG) to a protein is called “pegylation’. PEG moieties vary considerably in molecular weight and conformation [39]. The objectives of pegylation of a therapeutic protein is to increase half-life and decrease clearance, lower toxicity, and increase drug stability and solubility. 

Pegylation on ADCs as a linker may be helpful in improving the solubility and reducing the aggregation of ADCs. A study by Burke et al. [40] evaluated the impact of PEG side chains on the pharmacokinetics of monomethylauristatin E (MMAE). Multiple PEG-glucuronide-MMAE linkers were prepared with PEG size ranging from 2 to 24 ethylene oxide units, and each ADC was conjugated with eight MMAE drug molecules. Rats were given 3 mg/kg MMAE intravenously. The results of the study showed that longer PEG chains resulted in slower clearance. However, clearance remained unaltered with a threshold length of PEG_8_. This indicated that pegylation could be useful to enhance the PK and therapeutic efficacy of ADCs.

## 9. Size and Charge

Therapeutic proteins (monoclonal and polyclonal antibodies and non-antibodies) vary in size, which can have an impact on the PK of these macromolecules. For example, IgGs are much larger in size (at least three times) than Fab fragments. A PK study in mice indicated that the clearance of Fab fragments was 10 times higher than IgGs [41].

The charge of a protein is an important factor that can influence the PK of a protein. The isoelectric point (IP) is between 8 and 9, at which a protein carries no net electrical charge. According to Boswell et al. [42], even a shift of one IP unit can impact the PK and tissue distribution of a protein. Furthermore, an increase in net-positive IP results in increased tissue retention and a decrease in net-positive charge leads to decreased tissue retention.

At the moment, there is not much information regarding the impact of charge on the ADCs but it must be considered during ADC’s manufacturing process.

## 10. Linkers

Linkers are an integral part of ADCs. The linkers dictate the drug release mechanism, PK, therapeutic index, and safety of an ADC. The linkers provide the stability of drug antibody conjugation in the circulation. Early generation ADC linkers were chemically labile linkers such as disulfides and hydrazones [43,44,45,46,47]. These linkers were unstable in the circulation with short half-lives, generally one to two days. The recent generation of linkers are more stable in the systemic circulation such as peptide and glucuronide linkers. Peptide-based linkers comparatively provide better stability and drug release. The two most common linkers are as follows [43,44,45,46,47]:

- Cleavable linkers are peptide-based linkers and are cleaved by cathepsin B
- Non-cleavable linkers are thioether linkers that release the drug after the monoclonal antibody is degraded.

### 10.1. Non-Cleavable Linkers

Non-cleavable linkers are an important component of ADCs [43,44,45]. Non-cleavable linkers are a new generation of linkers with improved plasma stability compared to cleavable linkers. The ADCs prepared by non-cleavable linkers rely on the complete lysosomal proteolytic degradation of the antibody after internalization to release the cytotoxic small molecule drug. The ADCs with non-cleavable linkers are dependent on the biology of the target cell compared to cleavable linkers and have an improved therapeutic index due to their greater plasma stability. Since non-cleavable linkers can provide greater stability and tolerance than cleavable linkers, in the circulation, these linkers reduce off-target toxicity and also provide a larger therapeutic window [44,45,46].

### 10.2. Cleavable Linkers

Cleavable linkers play a critical part in the design of antibody–drug conjugates. They are safe in the blood circulation for an extensive period of time and effectively release their cytotoxic payload in the tumor microenvironment for removal of the tumor cells [43,44,45]. The most commonly used non-cleavable linkers in antibody-drug conjugates are succinimide–thioether bonds, which are formed by the reaction of maleimides with thiols [43].

Several other forms of linkers [46,47,48,49] such as chemically labile linkers (hydrazone and disulfide), acid-cleavable linkers (hydrazone), reducible linkers, peptide-based linkers, and β-Glucuronide linkers are also part of ADCs’ linker conjugation.

## 11. Allometric Interspecies Scaling of ADCs:

Allometric scaling is a useful tool for the extrapolation of pharmacokinetic parameters from animals to humans. This process, known as ‘interspecies scaling’, is widely used to predict human PK parameters [50]. These predicted PK parameters can then be used to design a first-in-human dose selection. Allometric interspecies scaling is applied to both small and large molecules (therapeutic proteins that includes monoclonal antibodies and non-antibodies) and a lot of work has been carried out in this direction [51,52,53,54]. Where a lot of information on the interspecies allometric scaling of small as well as large molecules are available, not many studies related to the interspecies scaling of ADCs are available. This is mainly because preclinical ADCs data are not readily available. Nevertheless, recently, some efforts were made to apply interspecies allometric scaling to predict human PK parameters, mainly clearance and volume of distribution from preclinical PK data.

Li et al. [55] predicted human clearance of ADCs from preclinical data. The authors used a multiple- as well as a single-species allometric scaling approach. In the analysis, there were six ADCs with two animal species and two ADCs with three animal species and the predicted human clearance was compared with the observed human clearance. Based on their analysis, the authors concluded that the monkey alone provided better results than the multiple-species scaling (within 0.5- to 2-fold).

In another study, Mahmood [56] predicted human clearance of ADCs using interspecies allometric scaling from one to three animal species and compared the predicted human clearance with the observed human clearance. In his study, Mahmood expanded one-species scaling analysis beyond monkey. It was noted that the exponent 1.0 (as used by Li et al.) provided comparable results between monkey and rat but the prediction of the clearance of ADCs was not as accurate for mouse, as noted with rat or monkey. In order to improve the predictive performance of one-species scaling, Mahmood applied three different exponents (0.80, 1.0, and 1.1) and then the average of predicted human clearance was taken and compared with the observed human clearance. This approach did not improve the human clearance prediction from rat or monkey, but it substantially improved human clearance prediction from mouse. For a single species scaling, when a fixed exponent 1.0 was used 80%, 71%, and 60% predicted human clearance values were within a 0.5- to 2-fold prediction error from monkey, rat, and mouse, respectively. When a multiple exponent and average method was applied to the mouse clearance, 80% predicted human clearance was within 0.5- to 2-fold prediction error.

Overall, the predicted human clearance values of ADCs from preclinical species was good. The allometric methods proposed by Li et al. and Mahmood can be used to predict human clearance from the animal data and subsequently to select the first-in-human dose of ADCs. Allometric scaling as compared with other empirical methods is not only simple but is robust and accurate enough to predict human PK parameters from preclinical species and then subsequently use for the selection of first-in-human ADC dose.

## 12. Imuunogenicity

Therapeutic proteins induce immune response following their administration to animals and humans. This immune response, termed ‘immunogenicity’, has the potential to impact the pharmacokinetics, pharmacodynamics, safety, and efficacy of a therapeutic protein. The impact of immune response on the clinical efficacy of therapeutic proteins in a patient population are generally variable, ranging from no measurable effect to serious side effects. The detection of anti-drug antibody (ADA) of a therapeutic protein in early drug development in clinical settings is of the utmost importance since the formation of ADA can not only compromise the efficacy of the product but can also produce undesirable effects. Therefore, in order to detect ADA of therapeutic proteins, it is essential to develop valid, sensitive, specific, and selective assays to measure ADA responses [57,58,59].

The FDA package inserts of ADCs advocate the following, and it should be considered during the development of ADCs and the interpretation of immunogenicity data: “Immunogenicity data are highly dependent on the sensitivity and specificity of the test methods used. Additionally, the observed incidence of a positive result in a test method may be influenced by several factors, including sample handling, timing of sample collection, drug interference, concomitant medication and the underlying disease. Therefore, comparison of the incidence of antibodies to a product with the incidence of antibodies to other products may be misleading”.

Carrasco-Triguero et al. [60] reported a study in which Genentech evaluated the immunogenicity of eight ADCs. These ADCs had the same conjugation chemistry and valine-citrulline-MMAE (vc-MMAE) linker drug. For each ADC, ADA prevalence at baseline, incidence, after treatment and other characteristics of the immune response (persistent or transient nature, onset) were evaluated. The immunogenicity assays used biotin and digoxigenin (DIG)-ADC conjugated reagents. Phase I and phase II clinical studies with eight ADCs were used in this assessment. The ADCs were administered every three weeks as single agents or in combination with other therapeutics. The phase I studies were based on dose escalation and expansion phases with primary objectives to evaluate the safety and to inform dosing for phase II and phase Ib combination studies. The secondary objectives were to evaluate PK and immunogenicity characteristics of these ADCs. Phase II primary objectives were safety and efficacy, and the secondary objectives were PK and immunogenicity characterization. Serum samples for immunogenicity analysis were collected at baseline before starting treatment with the ADCs and post-baseline prior to re-dosing during the treatment with the ADC in cycles 1 through 4, at the end of treatment, early termination, discontinuation and after 16 cycles. The concentrations of the total antibody were measured at all the ADA sampling time points. The results of this study can be summarized as follows:

The baseline prevalence of ADAs ranged between 1.4 and 8.1%. The post-baseline incidence of ADAs ranged between 0 and 35.8%. According to the authors, these values were within the range of therapeutic mAbs. In general, fewer patients developed ADAs with ADCs targeting hematological tumors than with those targeting solid tumors. The majority of the ADAs were against the mAb domain of the ADC, with a range between 86 and 100%. According to the authors, the results of the study indicated that in most patients the hapten-like structure of these ADCs did not produce more immune response risk than therapeutic mAbs.

## 13. Pharmacokinetics of ADCs in Children

Dosing of a drug is a critical path of drug development not only for adults but in children also. Many clinical trials fail due to poor dose selection [61,62]. Drug development for pediatrics is important because pediatric diseases may differ from those of the adults in terms of etiology, mechanisms, clinical or biological features, and the course of disease. The pharmacokinetics and pharmacodynamics of drugs, in most instances, are different in children than adults [63].

In recent years with the advent of pediatric exclusivity and requirements to conduct clinical studies in children, there has been greater emphasis to conduct safety and efficacy as well in clinical pharmacology studies in children. The Food and Drug Administration Amendments Act of 2007 reauthorized the Best Pharmaceuticals for Children Act (BPCA) and the Pediatric Research Equity Act (PREA). Both of these are designed to encourage more research and development for children’s health [64].

Besides small molecules, clinical trials for dosing of macromolecules such as monoclonal and polyclonal antibodies, non-antibodies, and coagulation factors in children are regularly conducted [65,66,67]. However, pediatric clinical trials have not been conducted for many approved ADCs. PK data of ADCs are scarce in pediatrics. In this review, the PK information for two ADCs in children are provided.

### 13.1. Pharmacokinetics of Gemtuzumab Ozogamicin (GO) in Children

Gemtuzumab ozogamicin is for the treatment of CD33-positive acute myeloid leukemia (AML) in first relapse in patients older than 60 years of age. Buckwalter et al. [68] studied the PK of GO in pediatric patients with relapsed or refractory AML. The study was a multicenter, dose-escalation, open-label, two-part outpatient study with an observation period following administration of GO. The patients received two doses of GO as a 2-h intravenous infusion separated by 14 to 28 days. Dosages of 6, 7.5, and 9 mg/m^2^ were administered during the study. Blood samples were drawn before the infusion, 1, 2, 3, 4, and 6 h post-infusion, and on study days 3, 5, and 10 of each dosing period. The children’s age and weight ranged from 1 to 16 years and 9.9 to 108.4 kg. Plasma samples were analyzed for hP67.6 (antibody) and calicheamicin derivatives (total and unconjugated) using validated enzyme-linked immunosorbent assay (ELISA) methods. PK parameters were estimated by non-compartmental analysis.

In Table 3, the PK parameters of hP67.6 (clearance (CL), volume of distribution at steady state (Vss), and half-life) as a function of age (period 1) are summarized. The CL and V_ss_ of hP67.6 increased as age increased and the half-life in infants (zero to two years) was longer than older children, adolescents, and adults. The PK parameters of hP67.6 in infants and children (3 to 11 years) were different than adolescents and adults (Table 3). The clearance of hP67.6 in infants and children was much slower than adolescents and adults. However, the sample size is too small to make any concrete conclusions.

The AUC and half-life of hP67.6, total calicheamicin, and unconjugated calicheamicin following 9 mg/m^2^ GO dose in period 1 were 136 ± 107, 3.15 ± 2.13, 0.261 ± 0.171 mg·h/L, and 63.7 ± 44.3, 42.0 ± 25.9, and 41.6 ± 50.0 h, respectively. The AUC and half-life of hP67.6, total calicheamicin, and unconjugated calicheamicin following 9 mg/m^2^ GO dose in period 2 were 241 ± 157, 6.85 ± 4.60, 0.499 ± 0.279 mg·h/L, and 57.8 ± 33.4, 59.0 ± 27.4, and 63.6 ± 44.2 h, respectively. These PK data suggest that the highest concentrations were observed for hP67.6 followed by total calicheamicin, and then the unconjugated form of ADC, substantially lower than hP67.6 and total calicheamicin. The concentrations of all moieties increased after the second dose. Changes in the concentrations of different moieties of GO following the second dose may be due to the saturation of CD33 binding sites and reduced tumor burden following the first dose [54].

### 13.2. Pharmacokinetics of Brentuximab Vedotin (BV) in Children

Flerlage et al. [69] studied the PK of BV in 16 children (4 to 18 years of age) with Hodgkin lymphoma. There was one subject between 6 and 10 years of age, four subjects 11 to 15 years of age, and 11 subjects 16 to 18 years of age. The authors reported that the AUC and C_max_ of BV were lower in pediatrics by 25% and 11%, respectively, as compared with adults. Most of the subjects were adolescents and it is not surprising that there is barely any difference in AUC and C_max_ of BV in this pediatric population.

The greatest impact of age on the PK of a drug (small or large molecules) is on the younger age groups, generally, ≤5 years of age. A general stratification of age groups is ≤five years (may be stratified within two age groups: ≤two years and >two to five years), >five to 12 years and >12 to <18 years of age [63,65].

## 14. Statistical Empirical Modeling of ADCs’ Pharmacokinetics and Pharmacodynamics

### 14.1. Population Pharmacokinetics (POPPK)

Besides non-compartmental analysis, compartmental analysis is also carried out to estimate PK parameters. However, both these methods require multiple blood samples (at least five to eight samples). The advantage of these two methods is that one does not need a large population size and a small sample size of four to six subjects is enough to obtain PK parameter estimates of a compound. However, as the clinical studies progress, the number of subjects increase, and it is not possible to take many blood samples from every subject. Therefore, a sparse blood sampling scheme (two to three blood samples) is generally used. Since the study population is large, it contains a lot of diversity, termed as ‘covariates’ such as body weight, gender, age, disease states (renal and/or hepatic impairment), drug–drug interaction studies, etc. The ultimate objective of a POPPK study is to determine the impact of the aforementioned or other relevant covariates on the PK and/or PD of a drug so that an optimum dose of a drug in a given patient population can be selected [70,71,72].

The caveats of POPPK studies should also be noted. Due to sparse sampling, model misspecification can be an issue for accurate estimation of PK parameters. POPPK studies are rarely validated by external data and a POPPK model is considered accurate based on statistical criteria. It should be recognized that many POPPK models may not be applied to a different population or may have poor predictive power outside the range of data from which the POPPK model was developed [73].

For several ADCs, the PK parameters were estimated using a POPPK approach [12,74,75,76,77,78,79,80]. When conducting a POPPK study, one should recognize the complexities of ADCs. Reporting clearance and volume of distribution of an ADC for a moiety whose exact dose is not known is incorrect. For example, one should not report the clearance and volume of distribution of unconjugated moiety of ADCs because the dose of unconjugated moiety is not known. It was noted that several POPPK studies reported the clearance of unconjugated moiety [77,79] and some authors [80] only reported the C_max_ and AUC, a correct approach.

Another critical issue in the POPPK analysis is related to the use of the fixed allometric exponents or slopes (0.75 for clearance and 1.0 for volume of distribution). It is widely believed by the modelers that the exponents 0.75 and 1.0 are biological or physiological. Allometry is nothing to do with the biological or physical system. Allometry is a mathematical manipulation to linearize non-linear data for ease of calculation. Allometry can be applied to both biological and physical systems. Most of the physiological or biological parameters are allometrically related but it does not make allometric exponent(s) biological. The allometric exponents widely vary and are data-dependent [81].

Incorrect application of allometry such as choosing theoretical fixed exponents of 0.75 for clearance and 1.0 for volume of distribution without taking data into consideration may give incorrect estimates of PK parameters. There is no logical and scientific reason to believe that a particular concentration-time data will always have the same fixed exponents. It is not always necessary to apply allometry in the POPPK analysis. If a covariate (generally it is body weight on clearance and volume of distribution) is not wide enough, then allometry should not be applied because the data cannot be described allometrically.

In a POPPK study, Lu et al. [75] determined the exponents of allometry rather than fixing it for clearance and volume of distribution. The authors found that the exponents of allometry for clearance and volume of distribution were 0.49 and 0.596, respectively. Both these exponents were further off than the theoretical exponents of 0.75 and 1 for clearance and volume of distribution, respectively. More such studies will reveal the lack of reconciliation between the theoretical allometric exponent and the data-dependent exponent.

### 14.2. Pharmacodynamics (PD)

The PK parameters can be linked to the time course of pharmacological response [7]. The term pharmacodynamics (PD) can be described as the relationship between drug concentrations at the site of action or receptor(s) and a pharmacologic response (therapeutic and/or toxicologic) [7]. The PD of both small and large molecules are well-characterized and are regularly conducted.

As with PK, the PD of ADCs are also complex, and the PK/PD relationship of ADCs is not as straightforward as small or other large molecules. Many factors include concentrations of the mAb and ADCs in the central and peripheral compartments, cytotoxic agent, dissociation constant between the cytotoxic agent and the mAb outside or inside the tumor, and the ratio between cytotoxic agent and mAb [8]. The requirements of these several parameters for establishing a PK/PD relationship for the ADCs make it very difficult to develop a PK/PD model that is a true representative of the PK/PD relationship of the ADCs. At the moment, a thorough knowledge of the PK/PD relationship of ADCs is lacking [8].

### 14.3. Exposure–Response Relationship

Establishment of response in terms of efficacy or toxicity with exposure (mainly AUC) can be beneficial for determining a therapeutic or toxic dose of a product. In several studies, the exposure–response relationship for ADCs [78,82,83,84] were explored and useful information in terms of safety and efficacy was observed. The study of Li et al. [83] represents a simple and robust approach for the exposure–response relationship of ADCs and is summarized below.

#### 14.3.1. vc-MMAE ADCs

Li et al. [83] investigated the exposure–response relationship of eight vc-MMAE ADCs against different targets and for different tumor indications using data from eight first-in-human phase 1 studies. vc-MMAE antibody-drug conjugates (ADCs) consist of a monoclonal antibody (mAb) covalently bound with a potent anti-mitotic toxin (MMAE) through a protease-labile valine-citrulline linker. PK parameters (first dose of 2.4 mg/kg) of conjugated antibody (acMMAE), total antibody, and unconjugated MMAE were estimated using non-compartmental analysis and were compared across the eight vc-MMAE ADCs. Exposure–response relationships were explored with efficacy (objective response rate (ORR)) and safety (grade 2+ peripheral neuropathy) endpoints. acMMAE exposure was strongly correlated with total antibody exposure for all the eight ADCs; as a result, the authors only used acMMAE exposure in their exposure–response analysis. A strong correlation between acMMAE and unconjugated MMAE exposure was not observed. AUC_(0-infinity)_ at cycle 1 for acMMAE and unconjugated MMAE analytes were used as the exposure metrics and logistic regression was used to explore the exposure–response relationship. The exposure–response relationship was explored for four ADCs. A statistically significant (*p* < 0.05) exposure–response relationship was observed between acMMAE cycle 1 AUC_(0-infinity)_ and ORR for three out of four ADCs. On the other hand, there was no statistically significant positive relationship between unconjugated MMAE exposure and ORR (*p* > 0.05). The exposure–safety (grade 2+ peripheral neuropathy) relationship was found to be statistically significant, resulting in dose reduction and discontinuation of the ADCs. The exposure–safety relationship was not statistically significant with unconjugated MMAE.

The exposure–response relationship is an effective tool to establish safety and efficacy for a drug and is widely used for small molecules as well as large molecules but is not as extensive as small molecules. The concept can be extended to the ADCs and it is being used in assessing the safety and efficacy of ADCs. However, it should be recognized that, structurally, ADCs are much more complex than small or other large molecules. The choice of an ADC moiety in the exposure–response relationship will play a very important role. From the exposure–response studies conducted so far for ADCs, it seems that total or conjugated antibody compared with unconjugated is a better choice. The reason being that the difficulty in measuring the concentrations of unconjugated antibody accurately, which can have an impact on the accurate estimation of PK parameters, and then, the choice of a PK parameter such as AUC or C_max_. AUC, which represents the entire concentration–time curve, may be a better choice than C_max_. C_max_ as a single data point may not even be a true C_max_ because C_max_ is sampling-dependent. Furthermore, one should also recognize that model-dependent simulation to obtain AUC and C_max_ may carry some errors, which may have an impact on the exposure–response relationship. Li et al.’s approach [83] to establish the exposure–response relationship of vc-MMAE ADCs is a robust and simple method (non-compartmental PK analysis and logistic regression). Complex models do not necessarily provide better results than simple models.

In short, if the exposure–response relationship is developed with a proper understanding of the choice of an ADC moiety and the PK parameter(s), then practical and reliable information regarding the safety and efficacy of ADCs can be obtained.

#### 14.3.2. Physiologically Based Pharmacokinetic Models (PBPK)

Besides POPPK and other PK/PD approaches, another empirical model is PBPK. PBPK is widely used for the characterization of PK and drug–drug interaction studies for small and large molecules. PBPK models have been developed for several ADCs [85,86,87,88]. Considering the complexities of an ADC molecule, the predictive power of PBPK models requires extensive validation from external data (observed data) before its use can be justified for ADCs. It is also important to establish if a whole body or minimal PBPK [88] model is needed for ADCs. In short, modeling and simulation is a helpful tool to obtain relevant information regarding ADCs before clinical trials are initiated.

One should also recognize that the models are inherently erratic and making a model complex will not necessarily improve the predictive performance of a given model. According to George Box, “Since all models are wrong the scientist cannot obtain a “correct” one by excessive elaboration. On the contrary following William of Occam he should seek an economical description of natural phenomena” [89].

## 15. Conclusions

This review provides a summary of the advances and challenges in the development of ADCs. At the moment, the focus is on the development of ADCs in the area of oncology but there is a potential to extend the therapeutic benefits of ADCs to other disease areas. New research in the field of ADC is emphasizing the choice of appropriate antibodies, antibody conjugation, linkers and the associated chemistry, and payloads. These are integral parts of the development of ADCs and clinical pharmacology is going to play a major role in promoting the safety and efficacy of ADCs by providing the optimum dose to the patient population. As with other drugs, dosing of ADCs depends on many factors such as age, weight, gender, disease states (hepatic and/or renal impairment), and concomitant drug administration. Clinical pharmacology is a powerful tool for the identification of these factors and subsequently helping in finding an optimum dose for an individual or a particular group of patients.

## Figures and Tables

**Table 1:** FDA approved ADCs and their characteristics.

Trade Name	Generic Name	Conjugate	Average DAR	Indication	Target
MYLOTARG	Gemtuzumab ozogamicin	Calicheamicin	2–3	Hematological	CD33
ADCETRIS	brentuximab vedotin	Monomethyl auristatin E (MMAE)	4	Hematological	CD30
BESPONSA	Inotuzumab ozogamicin	Calicheamicin	6	Hematological	CD22
POLIVY	Polatuzumab vedotin	Monomethyl auristatin E (MMAE)	3.5	Hematological	CD79b
KADCYLA	Trastuzumab emtansine	Myatansinoid (DM1)	3.5	Solid tumor	HER2
ENHERTU	Trastuzumab deruxtecan	Deruxtecan (Dxd)	7–8	Solid tumor	HER2
PADCEV	Enfortumab vedotin	Monomethyl auristatin E (MMAE)	3.8	Solid tumor	Nectin-4
TRODELVY	Sacituzumab govitecan	Govitecan SN-38	7.6	Solid tumor	Trop-2
BLENREP	Belantamab mafodotin	microtubule inhibitor MMAF	4	Myeloma	BCMA

Taken and modified from reference [11].

**Table 2:** Pharmacokinetic parameters of FDA approved ADCs (only the antibodies).

ADCs	Dose	CL (mL/h)	V_ss_ or V_c_ (L)	Half-Life (Days)
MYLOTARG	9 mg/m^2^	350	21	2.6
ADCETRIS	0.1–3.4 mg/kg	65	6–10	4–6
BESPONSA	1.2–1.8 mg/m^2^	33	12	12.3
POLIVY	1.8 mg/kg	900	3.2 *	12
KADCYLA	3.6 mg/kg	680	3.1 *	4
ENHERTU	3.2–8 mg/kg	420	2.8 *	5.7
PADCEV	1.25 mg/kg	100	11	3.4
TRODELVY	10 mg/kg	140	3.2	16 h
BLENREP	2.5 mg/kg	900	11	12

* Volume of distribution of the central compartment. Compiled from the FDA package inserts and references [12,13].

**Table 3:** PK parameters (mean ± sd) of Gemtuzumab Ozogamicin in children following 9 mg/m^2^ in period 1 as a function of age.

Age (Years)	CL (L/h)	V_ss_ (L)	Half-Life (h)
Infants (0–2), *n* = 2	0.03 ± NA	2.9 ± NA	113 ± NA
Children (3–11), *n* = 5	0.06 ± 0.03	3.9 ± 1.9	45.6 ± 30.8
Adolescents (12–16), *n* = 7	0.26 ± 0.30	9.4 ± 6.6	62.0 ± 16.5
Adults (*n* = 59)	0.27 ± 0.23	20.9 ± 21.5	72.4 ± 42.0

NA because *n* = 2.

## Data Availability

Data were taken from the references provided in the manuscript.
